# When inflammation meets lung development—an update on the pathogenesis of bronchopulmonary dysplasia

**DOI:** 10.1186/s40348-022-00137-z

**Published:** 2022-04-20

**Authors:** Lena Holzfurtner, Tayyab Shahzad, Ying Dong, Lisa Rekers, Ariane Selting, Birte Staude, Tina Lauer, Annesuse Schmidt, Stefano Rivetti, Klaus-Peter Zimmer, Judith Behnke, Saverio Bellusci, Harald Ehrhardt

**Affiliations:** 1 Department of General Pediatrics and Neonatology, Universities of Giessen and Marburg Lung Center (UGMLC), Member of the German Lung Research Center (DZL), Justus-Liebig-University, Feulgenstrasse 12, 35392 Giessen, Germany; 2 Department of Internal Medicine II, Universities of Giessen and Marburg Lung Center (UGMLC), Cardiopulmonary Institute (CPI), Member of the German Center for Lung Research (DZL), Justus-Liebig-University, Aulweg 130, 35392 Giessen, Germany

**Keywords:** Chronic lung disease, Bronchopulmonary dysplasia, Preterm, Reactive oxygen species, Inflammation, Lung injury, Rodent, Therapeutic approach

## Abstract

Even more than 50 years after its initial description, bronchopulmonary dysplasia (BPD) remains one of the most important and lifelong sequelae following premature birth. Tremendous efforts have been undertaken since then to reduce this ever-increasing disease burden but a therapeutic breakthrough preventing BPD is still not in sight. The inflammatory response provoked in the immature lung is a key driver of distorted lung development and impacts the formation of alveolar, mesenchymal, and vascular structures during a particularly vulnerable time-period. During the last 5 years, new scientific insights have led to an improved pathomechanistic understanding of BPD origins and disease drivers. Within the framework of current scientific progress, concepts involving disruption of the balance of key inflammatory and lung growth promoting pathways by various stimuli, take center stage. Still today, the number of efficient therapeutics available to prevent BPD is limited to a few, well-established pharmacological interventions including postnatal corticosteroids, early caffeine administration, and vitamin A. Recent advances in the clinical care of infants in the neonatal intensive care unit (NICU) have led to improvements in survival without a consistent reduction in the incidence of BPD. Our update provides latest insights from both preclinical models and clinical cohort studies and describes novel approaches to prevent BPD.

## Introduction

More than 50 years ago, bronchopulmonary dysplasia (BPD) was first described by Northway, Rosan, and Porter [1]. Even after more than five decades of scientific progress, the overall BPD disease burden is high in the newborn population but a dramatic shift in infants at high risk and BPD pathology occurred since the original publication. Although in the past, even comparatively mature preterm infants were vulnerable to the development of lung emphysema, fibrosis, and high mortality characteristic of the “old” BPD, the implementation of antenatal steroids and postnatal surfactant therapy in the 1980s and 1990s has led to both improved outcomes for late preterm infants and the increasing survival of infants born as early as 22–24 gestational age. This so-called new BPD is characterized by less severe tissue injury, but more or less distinct disturbance of further lung development in the late canalicular and saccular stage [2].

Large cohort studies have demonstrated that low gestational age at birth and low birthweight [3] constitute major determinants of the risk for BPD; however, genetic predisposition [4], including male sex and maternal disorders including pre-eclampsia/HELLP (hemolysis, elevated liver enzyme levels, low platelet count), intrauterine growth restriction [5], and nicotine consumption [6] also impact on the likelihood to develop BPD. Besides the need for mechanical ventilation and oxygen supplementation, intrauterine and postnatal infections nowadays constitute important contributors to BPD. The presence of a patent ductus arteriosus and NICU management variations including fluid and nutritional supply, antibiotic regimens, and measures to prevent postnatal infections and necrotizing enterocolitis (NEC) including the provision of human milk constitute further variables impacting on the risk for BPD [2, 7–9].

**Fig. 1:**
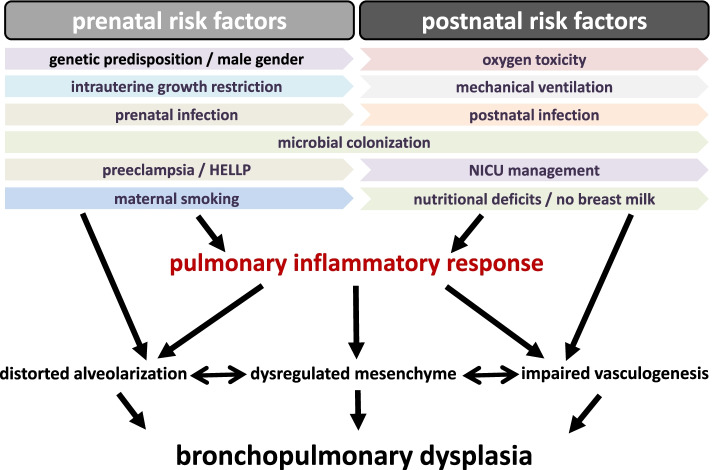
Central pre- and postnatal risk factors for BPD development. The key contributors to the development of BPD are split into prenatal and postnatal risk factors (scheme developed from our previous review on the topic [12])

Nowadays, the gas exchange of most preterm infants can be stabilized after birth but most of these tiny infants experience a more or less dramatic deterioration of their lung function requiring escalation of respiratory support and oxygen fractions applied [10]. It is well accepted that the respiratory course is determined by the extent of the inflammatory response in the immature lung provoked or strengthened by the multiple pre- and postnatal risk factors and pro-inflammatory stimuli [10–12]. The ever-increasing number of publications on BPD reflects the need for further gain in knowledge [13]. It was already elaborated in the original description of BPD that all compartments of the lung, the epithelium, the endothelium, and the mesenchyme get affected during the evolution of BPD pathology. This leads to a reduced surface for gas exchange, rarefication of pulmonary vessels, and pathologic composition of the lung interstitium [1]. Therefore, addressing the BPD challenge requires a comprehensive pathomechanistic understanding presented within the second chapter of our review and needs to acknowledge the multifactorial origins of BPD summarized in Fig. 1.

## The pulmonary inflammatory response

**Fig. 2:**
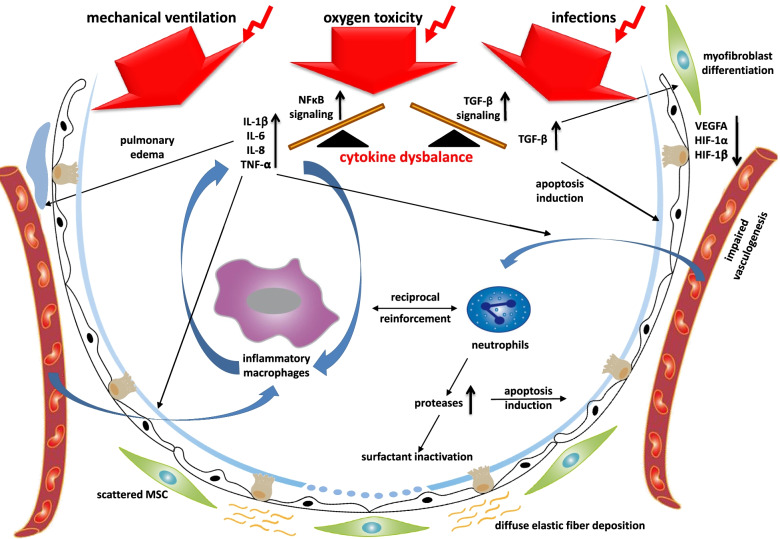
The inflammatory response in the immature lung as key event leading to bronchopulmonary dysplasia. Mechanical ventilation, oxygen supply and infections induce a pro-inflammatory response in the immature lung. This pro-inflammatory overweight disrupts the balanced network of growth signal pathways and leads to characteristic features in the lung that affect the epithelium, the mesenchyme and the endothelium (scheme developed from our previous review on the topic [12])

Within the last two decades, the general concept of inflammation of the immature lung leading to BPD in preterm infants has not been changed (Fig. 2). Results obtained from various experimental models of BPD have demonstrated that infectious insults, oxygen toxicity, and mechanical ventilation cause the characteristic pathological features of BPD with distorted alveolar and vascular growth and unfavorable changes in the composition of the mesenchyme and mesenchymal cell transdifferentiation [14]. In rodents studied using a model of hyperoxia-induced BPD, the extent of lung developmental aberrations is determined by the fraction and duration of oxygen supplementation [15]. BPD pathology gets aggravated when hyperoxic episodes are combined with hypoxemic events that frequently occur in preterm infants and further boost reactive oxygen species production and subsequent inflammation [16].

### Dysbalance of pro-inflammatory cytokines and of lung growth promoting factors

The inflammatory reaction is characterized by the excess production of pro-inflammatory cytokines including interleukin-1β (IL-1ß), interleukin-6 (IL-6), interleukin-8 (IL-8) and tumor necrosis factor-α (TNF-α), granulocyte colony-stimulating factor, macrophage inflammatory proteins, and monocyte chemoattractant proteins while anti-inflammatory cytokines like interleukin-10 get downregulated [12, 17]. Recent data from a study in very preterm infants indicate that the inflammatory response commences early after birth and precedes the deterioration of lung function [17]. For transforming growth factor-β (TGF-β), excess production was ascribed to pro-inflammatory activity and apoptosis induction but anti-inflammatory and immunosuppressive functions as well as growth promoting functions have also been confirmed [16, 18]. Further complexity arises when the inflammatory stimulus is not investigated within single hit models that do not reflect the clinical scenario. Either pretreatment with antenatal steroids or pre-exposure to ureaplasma species abrogated the intra-uterine inflammation induced by lipopolysaccharides [19, 20]. Therefore, the complexity of TGF-β signaling offers a prime example of how the role of a specific cytokine is not necessarily uniform and can be affected by both physiological and pathophysiological stimuli what will be detailed later in this section.

The overexpression of pro-inflammatory cytokines is counterbalanced by the suppression of growth promoting cytokines. Probably vascular endothelial growth factor A (VEGFA) constitutes the most prominent and best studied candidate for vascular development, while platelet-derived growth factor AA (PDGF-AA) was primarily related to mesenchymal stem cell (MSC) function and insulin like growth factor 1 (IGF-1) with alveolar epithelial and endothelial cells [21–23]. This cytokine and growth factor dysbalance is not restricted to the lung. Convincing evidence is available that describes a close correlation between pulmonary dysfunction and changes in systemic blood protein profile patterns [11].

### Attraction of innate immune cells to the immature lung

Upregulation in the expression of pro-inflammatory mediators attracts alveolar macrophages and neutrophils that constitute the prevailing leukocyte fractions of the innate immune response. The pro-inflammatory cytokines in the lung induce a switch from the initial anti-inflammatory M2 to the inflammatory M1 polarization status. The resulting predominance of the inflammatory M1 polarization status perpetuates and augments the inflammatory response among others by the release of further pro-inflammatory cytokines [24]. Neutrophils have also been shown to exert lung injury through the release of multiple proteases including metalloproteinases and elastase [12]. Neutrophil extracellular trap formation is one recently discovered hallmark leading to lung injury [25]. Furthermore, prevention of neutrophil influx following hyperoxia reduced reactive oxygen species (ROS) levels in the newborn rat lung and the severity of lung injury [26]. Besides macrophages and neutrophils, mast cells and reactive T cells were ascribed contributing functions to BPD pathology and it will be important to dissect the interplay between the different pro-inflammatory cell populations [12]. Plenty of observational data are available from biosample studies in preterm infants that were mostly performed around the turn of the century. The results of these studies have consistently demonstrated an association between excessive pro-inflammatory responses and suppression of growth factor signaling pathways [27]. Consultation of the upcoming and latest datasets from preclinical rodent models helps to further clarify the contribution of specific cytokines and targets to BPD pathology.

### Dysbalance of the cytokine signaling network in the immature lung

Major advances within the last 5 years have confirmed that precise and complex signaling crosstalk occurs between the three compartments of the lung during both physiological conditions and BPD development. Excessive activation of TGF-β signaling pathways leads to the suppression of PDGF receptor α mediated pulmonary myofibroblast function and VEGFA secretion. In this way, excessive inflammation is linked with dysregulation of vascular development [22]. This relationship can also be illustrated by the finding that disturbance in fibroblast growth factor 10 (FGF10) function is implicated in unfavorable alterations in alveolar epithelial cell properties that resulted in the reduction of type II cells in the lung and impaired production of surfactant proteins B and C [28]. The interconnection of FGF10 deficiency with improper lung vascular development was documented in the identical animal model with both rarefication of the number of blood vessels and abnormal muscularization noted. These findings are reflective of the pathological phenotype of infants with BPD associated pulmonary hypertension [29]. Furthermore, spatial and temporal regulations of growth signaling need to be considered when putting the focus on lung development in the late canalicular and saccular stage that is beyond the scope of our review. Further details and the available data on the further pathways including sonic hedgehog and Wnt signaling have been superbly detailed elsewhere [13].

### Phenotype distortion of lung resident mesenchymal stem cells

Although the essential role of lung MSCs in physiological lung development was identified as early as the 1990s, the therapeutic potential of exogenously applied umbilical cord derived MSCs has emerged as an exciting area of focus in BPD research [30]. All the studies on primary MSC from preterm infants confirmed the tight association between distorted lung resident mesenchymal stem cell phenotype and BPD [31, 32]. Again, the TGF-β and nuclear factor kappa-light-chain-enhancer of activated B cells (NFκB) signaling pathways were identified as key drivers of MSC pathology [32, 33]. As demonstrated, redirection of MSC phenotype alterations is in principle feasible but awaits further detailing in therapeutic intervention studies in in vivo models [32]. More sophisticated strategies than simply inhibiting the excessive NFκB response will be described later in this section.

### Dual function of inflammatory cytokine signaling pathways during lung development and injury

As stated above, more and more cytokines and pathways get discovered that both contribute to physiologic lung development but can aggravate lung injury when overstimulation occurs. Perhaps the longest known connection is that of TGF-β signaling. While Smad-3 knockout mice displayed a normal lung phenotype at birth, morphological changes characterized by enlarged terminal air spaces became apparent on postnatal day 7. In the term born mouse, this time point corresponds approximately to the early phase of alveolarization that begins between 34 and 36 weeks postmenstrual age (PMA) in humans [18, 34]. This is in line with the previous observation of dynamic regulation of TGF-β signaling in mice and men during lung development [35]. Excess in TGF-β in tracheal aspirates of ventilated preterm infants was associated with BPD and lung epithelial TGF-β1 overexpression led to arrested lung development in the rodent model [36, 37]. Further details were recently provided from mice with a mesenchyme-specific deletion of TGF-β1. Here, embryonic lung branching morphogenesis was disrupted resulting in lung hypoplasia [38]. Inversely, the hyperactivation of the connective tissue growth factor (CTGF)-β-catenin signaling pathway downstream of TGF-β1 by hyperoxia was associated with the characteristic features of BPD lungs. Inhibition of this axis by CTGF neutralizing antibodies or pharmacological inhibition of β-catenin both attenuated aberrations in alveolar and vascular lung development [39, 40].

Similarly, the disruption of NFκB signaling resulted in distortion of further alveolar and vascular lung development [41]. The detailed examination of NFκB action revealed that the inhibitor of nuclear factor kappa-B kinase beta (IKKβ) but not IKKα was the predominant regulator of angiogenic function and pulmonary angiogenesis [42]. In line, knockout mice for TNF-α displayed reduced NFκB activation when subjected to mechanical ventilation in moderate hyperoxia and more aggravated lung inflammation and apoptosis induction [18]. The essential role of NFκB to preserve the lung from injury was extended to the situation of lipopolysaccharide (LPS)-induced inflammation mimicking gram-negative infections. Here, inhibition of NFκB aggravated the suppression of alveolarization and pulmonary angiogenesis, a finding that was attributed to increased expression of macrophage inflammatory protein 2 (MIP-2). This mediator acted directly to inhibit pulmonary endothelial cell function, a step that initiated the aberration of normal lung development [43]. As for the hyperoxic injury, inhibition of IKKβ prevented the pro-inflammatory response of macrophages [44]. Further complexity on the topic arises from one recent publication on macrophage derived IL-6 signaling. Here, the loss of IL-6 was associated with better preserved alveolar epithelial type II cell survival and elastic fiber assembly while myofibroblast differentiation was reduced [45]. These findings are in contrast to those published by another group that reported a more pronounced inflammatory response and increased severity of lung injury in IL-6 knockout mice exposed to hyperoxia [46]. The results are in line with the knowledge on the diverse functions of IL-6 that can exert anti-inflammatory and reparative effects or pro-inflammatory responses depending on the signal transduction pathway [47]. This ambiguity on IL-6 accentuates the concerns that pro-inflammatory cytokines in general have divergent functions during lung injury. Further evaluation is urgently needed whether pro-inflammatory cytokine targeting can evolve as a novel safe therapeutic approach.

From all these studies, it comes clear that the pro-inflammatory cytokines that activate NFκB or TGF-β signaling contribute to proper lung development under physiologic conditions but aggravate lung injury under excess activation. The key event of MSC phenotype alterations in this context as the origin of aberrant further lung development comes more and more into the focus of researchers [32, 33]. The original concept to block pro-inflammatory activity to overcome the deleterious effects on lung development needs to be substituted by the more sophisticated approach to restitute the physiologic situation of a balanced signaling network where excess activation of any partner involved leads to dysregulation and aggravation of lung injury.

## Strategies to promote lung development and to attenuate the pro-inflammatory response

The tremendous progress in preclinical knowledge and the pathomechanistic understanding of the origins of BPD contrasts the factum that no novel therapeutic was added to the short list of medications to prevent BPD since our first review on the topic five years ago [12]. In this section, we will provide a short overview on key advances during the last 5 years and on actually ongoing research directions. For the level of statistical significance and confidence intervals, we refer the reader to the original publications.

### Therapeutic interventions of proven efficacy within clinical trials

Still today, the well-studied medical interventions are limited to postnatal corticosteroids and postnatal caffeine and vitamin A [16, 30]. The clinical trials on inhaled nitric oxide to prevent BPD more than 10 years ago were the last series testing a new medication to prevent BPD but failed to display a benefit on mortality, the pulmonary and neurodevelopmental outcome [48]. In addition, the combination of inhaled nitric oxide (iNO) together with repeated surfactant application did not demonstrate any benefit [49]. These completely negative results despite highly promising perspectives from preclinical studies might have discouraged researchers to pursue the direction toward new therapeutics. Results of a recent phase 2 randomized controlled trial designed to evaluate the potential of rhIGF-1/rhIGFBP3 administration to reduce retinopathy of prematurity (ROP) in preterm infants born before 28 weeks gestational age (GA) (*n* = 121) showed that although there was no significant reduction in the primary outcome, this intervention was associated with a significant reduction in the incidence of severe BPD [50]. These results are not surprising taking into account the observed reduction of IGF-1 levels in preterm infants after birth and during the first weeks of life and the beneficial effects of IGF-1 therapy in rodent studies on lung development [21, 51, 52]. Of course, this initial study needs confirmation in adequately powered randomized controlled trials and the mitogen functions of IGF-1 need critical monitoring as is currently intended within an open label controlled three-arm phase 2b study on safety, optimal dosing, and efficacy (NCT03253263).

### The avenue to further targeted interventions

Although the evidence for safety and efficacy is currently lacking, the results of the IGF-1/IGFBP3 pilot trial might be useful in encouraging the pursuit of therapeutic strategies involving lung growth promoting cytokines rather than previous approaches that were focused on the use of anti-inflammatory agents. During the recent years, several further candidates have been evaluated in detail in preclinical models. One of the most promising candidates was VEGFA, as disruption of pulmonary vessel formation is a hallmark of BPD pathology that was ascribed to the suppression of VEGFA. The application of VEGFA improved lung structures following hyperoxia but was accompanied by increased vessel leakage and pulmonary edema in the acute phase of injury. These results must be seen in the context of the physiologic function of VEGFA in vascular development that is to instigate branching of blood vessels by increasing permeability. Therefore, this activity needs to be counterbalanced by appropriate angiopoietin signaling that safeguards vascular integrity [53]. Confirming this interrelation of action, simultaneous application of angiopoietin-1 by gene transfer prevented the vascular vessel leakage [54, 55]. Further concerns about the therapeutic potential of modulating the hypoxia induced factor (HIF)-1α-VEGFA axis arose, when mice with stable overexpression of HIF-1α-subunit in the distal epithelium were exposed to hyperoxic injury. The stable overexpression of HIF-1α increased the levels of VEGFA family members and angiopoietins but failed to improve lung structures and resulted in poorer lung function [56]. Further promising growth factor candidates are currently thoroughly investigated for their therapeutic potential to prevent or treat BPD. The therapeutic potential of PDGF receptor-α signaling was confirmed in haploinsufficient mice where intrapulmonary treatment with PDGF-AA rescued the more pronounced lung injury by mechanical ventilation and oxygen toxicity [22]. Another highly promising candidate constitutes FGF-10. Detailed studies in transgenic newborn documented the beneficial effects on the lung epithelium and vessel formation [28, 29]. Research on further candidates comprise epidermal growth factor (EGF), keratinocyte growth factor (KGF) and hepatocyte growth factor (HGF) but are still at the onset of a thorough evaluation. One so far mostly neglected need is to establish not only preventive strategies but to document the therapeutic potential to counteract the arising pathologies of BPD as most preterm infants are already exposed to lung injuries in utero or shortly after birth.

Overall, the track to growth factor treatment to prevent or treat BPD is still a long journey maybe with the exception of IGF-1. It needs to be established whether the selective intervention into one cytokine pathway can be as effective as the broadly acting corticosteroids that are still today the choice of therapy to abrogate the inflammatory response in the immature lung provoked by oxygen, mechanical ventilation, and infection. Overall, postnatal corticosteroid application > 7 days of life are highly effective and at least for dexamethasone a clear benefit to prevent BPD at 36 weeks corrected age has been demonstrated [57]. In contrast, early corticosteroid application prior to 7 days of life poses dramatic risks for neurodevelopmental impairment and cerebral palsy and should therefore not be used [58, 59]. Administration of caffeine within the first week of life is another intervention with documented benefit to reduce the BPD burden that is supported by large scale randomized controlled trial data [60]. Although the action of caffeine in the prevention of BPD has been largely ascribed by clinicians to occur through the stabilization of respiratory drive [61], data from preclinical studies has indicated that benefits may also be derived through the antioxidant and anti-inflammatory properties of this now commonly used medication [62, 63]. These data are not surprising taking into account the contribution of ROS induction by infection and oxygen therapy to the proinflammatory response in the lungs of preterm infants and BPD [64–66].

### Infections, pathologic microbiota structures, and probiotics application

In the context of inflammation, nosocomial infections represent another entity that increase the risk for BPD and act via the identical pathomechanisms. On the other hand, exposure to the powerful anti-inflammatory and immunomodulatory properties of human milk constitutes a key non-pharmacological intervention suggested to reduce the incidence of BPD [67]. These data should be expected when the pathomechanisms of BPD are taken into consideration. One further dimension came into the focus of research during the recent years, the shape of the preterm’s microbiome. It is highly accepted that the provision of human milk shapes the bacterial milieu in the preterm infant toward *Bifidobacteria* and *Lactobacillus* species while the predominance of potentially pathogenic germs is impeded [9]. Therefore, human milk feedings may act to reduce the incidence of BPD by protecting infants from nosocomial infections and NEC. But even in the absence of infection events, the shift of the microbiota structures toward an anti-inflammatory milieu might contribute to the risk reduction by human milk. In line, antibiotic exposure with the selection of pathogenic bacterial species increases the risk of BPD in clinical studies [68, 69]. Recent data from a prospective randomized controlled trial specify the risk toward exposures longer than the first 2 days of life [70]. While germ-free mice were partially protected from injury caused by hyperoxia [71], exposure to ampicillin during the pre-natal and immediate post-natal period was found to be associated with increased severity of hyperoxia induced alveolar simplification and dysregulated vasculogenesis [72]. In contrast to the benefits for sepsis and necrotizing enterocolitis, probiotic bacteria administration did not result in any benefit for the lung so far although specific microbial compositions of tracheal aspirates were associated with the development of BPD independent of the occurrence of pneumonia [9, 73]. This discrepancy might be explained by the fact that probiotics fail to stabilize the microbial milieu in the upper airway that seems to be of greater relevance for the lung than that in the gut [7]. Although so far not marked by success, a healthy microbiome and modulation of the bacterial milieu in utero and in the preterm infant constitutes a highly promising approach taking into account the tremendous disease burden arising from prematurity until aging [74, 75]. Recent data indicate that the approach to the lung and the gut might need different strategies although both entities arise from the identical bud [7, 73, 75].

### Maternal risk factors and BPD

Altogether, these data indicate that external factors impact on the inflammatory milieu and the risk of BPD. Therefore, the focus on the environmental contributors might be another strategy toward efficient risk reduction for BPD. While diabetes in pregnancy does not increase the risk for BPD, maternal nicotine exposure does [76, 77]. Pre-pregnancy no smoking prevention programs and the avoidance of exposure of the preterm infant to the substances of tobacco smoke are further suited strategies. As intensification of smoking prevention programs will not terminate the intrauterine exposition, prenatal therapeutic interventions can constitute an additional approach to reduce the disease burden of BPD. Here, results from randomized controlled trials with prophylactic vitamin C supplementation for pregnant smoking women revealed improved pulmonary function and decreased wheezing episodes in mostly term born infants [78, 79].

Although a completely different pathomechanism with primary action on vascular development via vascular growth inhibiting factors, the prevention of preeclampsia/HELLP or strategies to prevent the injury by soluble Fms-like thyrosinkinase-1 (sFlt-1) constitute another area of perspective [80].

### Anti-inflammatory approaches with documented preclinical efficacy

Despite the long list of therapeutic strategies, that proved efficacious in rodent models but failed to translate into new efficient clinical therapeutics, it needs to be mentioned at this point that several anti-inflammatory strategies proved their potential as novel targeted therapies that deserve further evaluation within the preclinical setting [81]. Firstly, interleukin-1 receptor antagonists (IL-1Rα) reduced the overall pulmonary cellular inflammatory response and pro-inflammatory cytokine levels of IL-1β, IL-6, MIP-1α, MIP-1β, and MIP-2 [82]. Secondly, biochemical Ras-related C3 botulinum toxin substrate 1 (Rac-1) inhibition and the irreversible caspase-1 inhibitor Ac-YVAD-CMK both attenuated the inflammation-induced IL-1β release and inflammasome activity in the lung exposed to hyperoxia underlining the potential of such targeted approaches [83, 84]. Thirdly, targeting IL-6 release from inflammatory macrophages in the lung seems highly promising [45].

Targeting the pathologic processes downstream of the initiation of the inflammatory response constitutes another attractive approach toward prevention of lung injury. To give one example, elafin is the specific inhibitor of lung elastase that is a contributor to lung injury in the execution phase [85]. Either intrapulmonary elafin treatment in newborn mice or mice genetically modified to express elafin in their vascular endothelium were partially protected from the injurious insults and defective late lung development. Of notice, the pathologic activation of the pathways of TGF-β and NFκB, the influx of inflammatory cells, and apoptosis induction in the lung were partially prohibited [86, 87]. These data hint toward the potential success of strategies that aim at disrupting the vicious circle and self-reinforcement of inflammation. The continuation of studies on the therapeutic potential of elafin in newborn mice exposed to prolonged hyperoxia underlined its potential of repetitive application [88]. The results obtained from all experimental settings evaluating elafin are highly promising because they are consistent and reproducible. But VEGFA signaling and pulmonary vessel formation were not rescued by elafin treatment underlining the need for detailed studies on all three lung compartments when evaluating molecular mechanisms and targeted interventions [86].

Lastly, the upcoming research area on MSC to prevent BPD needs to be mentioned here. They are deemed to be particularly promising due to their broadly acting anti-inflammatory and growth promoting properties [30, 89]. Preclinical studies conducted using a variety of small rodent BPD models have demonstrated high therapeutic efficacy for MSC-based interventions even when treatment is applied after lung injury has occurred [24, 90]. MSC-derived exosomes (extracellular vesicles = EVs) are currently investigated as the next level of MSC research due to their lower immunogenicity and smaller size. These EVs contain all key anti-inflammatory and immunomodulatory mediators that explains their comparable efficacy to the direct MSC application [30]. One actual study in rodents further specified one main effect of EV action where EVs restored a non-inflammatory phenotype of lung myeloid cells by phenotypical and epigenetic reprogramming [91]. These data open the frame toward restoration of lung functional capacities even after intrauterine affection of the lung or immediate postnatal insults before a therapeutic intervention can be started. But as for many other therapies, the documentation of superiority in the clinical setting is still missing. Latest data from non-rodent models argue toward a careful consideration of aspects like cell preparation, timing, and dosing to reach finally a superiority in the clinical setting [30, 89]. The highly promising results obtained in rodents await confirmation in higher developed animal species like lambs and primates and clinical trials in preterm infants. So far, one phase 1 study intended for safety issues documented a benefit for BPD while the subsequent phase 2 study of the same group confirmed the safety but did not detect an effect on BPD [92, 93]. Future studies are ongoing that can advance the scientific knowledge of the therapeutic potential of MSC [94].

## Outlook and research directions in ongoing clinical trials

Over the last 5 years, we have seen a tremendous gain in knowledge on the pathomechanisms of BPD and the complexity of its disease origins. But on a short-term perspective, we do not expect novel targeted interventions to revolutionize clinical therapy as many hurdles including the successful conductance of randomized controlled multicenter studies will take at least several years or even a decade before a benefit for the lung can be established. In the meantime, the optimization of established therapeutics including antenatal steroids to reduce the severity of respiratory distress after birth and studies on established therapeutics like vitamin A remain most promising within the pharmacologic approaches [95]. One just published smaller randomized controlled trial on oral vitamin A instead of the established approach of intramuscular application delivered disappointing results as high dose vitamin A did not reduce the overall BPD incidence and BPD severity distribution despite improving vitamin A blood levels [96, 97]. Therefore, still today as demonstrated during the last decade, optimization of ventilatory strategies, non-invasive surfactant application, and oxygen provision remain most promising to guide future treatment directions in the short run [66, 98, 99]. Besides the avoidance of mechanical ventilation, the stabilization of infants within the oxygen saturation targets is another promising strategy as hyperoxic and hypoxic episodes both aggravate lung injury. Several randomized controlled trials have been started into this direction. The OPTTIMMAL study aims to find the positive end-expiratory pressure (PEEP) level that is best suited to avoid intubation and mechanical ventilation within the first days of life when the immature lung is particularly vulnerable [100]. As another example, the Fi02C study investigates the automatically controlled titration of oxygen fractions by the respirator based on the actual oxygen saturation of the preterm infant during the total phase of respiratory support [101]. Furthermore, studies like the COSGOD III trial and the SafeBoosC III trial on tissue oxygenation of the brain during resuscitation in the delivery room and during respiratory support in the NICU respectively were designed to provide novel insights into the optimization of tissue oxygen saturation monitoring [102, 103]. In this context, the recent secondary analysis from the Canadian oxygen trial provided novel insights onto the association of the number and duration of hypoxemic episodes of preterm infants during their NICU stay and the later development of severe BPD [104]. Due to the observational character of the analysis, this study was not able to clarify the mechanistic link between hypoxemic episodes and BPD. The increased number of events can just reflect the more severe injury status of the lung in these infants. Here, the Pre-Vent study aims to precise the effects and mechanisms of ventilatory control to the adverse respiratory outcome [105].

From all these studies, we await novel insights into the origins and causes of BPD and which research directions are the most promising to further optimize the ventilatory strategies in preterm infants to reduce the burden of BPD.

## Data Availability

Not applicable.

## Abbreviations

BPD: Bronchopulmonary dysplasia
CTGF: Connective tissue growth factor
EGF: Epidermal growth factor
EVs: Extracellular vesicles
FGF10: Fibroblast growth factor 10
GA: Gestational age
HELLP: Hemolysis, Elevated Liver Enzyme Levels, Low Platelet Count
HGF: Hepatocyte growth factor
HIF: Hypoxia induced factor
IGF-1: Insulin like growth factor 1
IKKα: Inhibitor of nuclear factor kappa-B kinase alpha
IKKβ: Inhibitor of nuclear factor kappa-B kinase beta
IL-6: Interleukin-6
IL-1Rα: Interleukin-1 receptor antagonists
IL-1β: Interleukin-1β
KGF: Keratinocyte growth factor
LPS: Lipopolysaccharide
MIP-1α: macrophage inflammatory protein 1α
MIP-2: Macrophage inflammatory protein 2
MIP-1β: Macrophage inflammatory protein 1β
MSC: Mesenchymal stem cell
NEC: Necrotizing enterocolitis
NFκB: Nuclear factor kappa-light-chain-enhancer of activated B cells
NICU: Neonatal intensive care unit
iNO: Inhaled nitric oxide
PDGF-AA: Platelet-derived growth factor AA
PEEP: Positive end-expiratory pressure
PMA: Postmenstrual age
Rac-1: Ras-related C3 botulinum toxin substrate 1
ROP: Retinopathy of prematurity
ROS: Reactive oxygen species
sFlt-1: Soluble Fms-like thyrosinkinase-1
TGF-β: Transforming growth factor-β
TNF-α: Tumor necrosis factor alpha
VEGFA: Vascular endothelial growth factor A
